# Executive function impairments in fibromyalgia syndrome: Relevance of clinical variables and body mass index

**DOI:** 10.1371/journal.pone.0196329

**Published:** 2018-04-25

**Authors:** Cristina Muñoz Ladrón de Guevara, María José Fernández-Serrano, Gustavo A. Reyes del Paso, Stefan Duschek

**Affiliations:** 1 Department of Psychology, University of Jaén, Jaén, Spain; 2 UMIT—University for Health Sciences Medical Informatics and Technology, Hall in Tirol, Austria; University Of São Paulo, BRAZIL

## Abstract

**Background:**

Several investigations suggest the presence of deterioration of executive function in fibromyalgia syndrome (FMS). The study quantified executive functions in patients with FMS. A wide array of functions was assessed, including updating, shifting and inhibition, as well as decision making and mental planning. Moreover, clinical variables were investigated as possible mediators of executive dysfunction, including pain severity, psychiatric comorbidity, medication and body mass index (BMI).

**Methods:**

Fifty-two FMS patients and 32 healthy controls completed a battery of 14 neuropsychological tests. Clinical interviews were conducted and the McGill Pain Questionnaire, Beck Depression Inventory, State-Trait Anxiety Inventory, Fatigue Severity Scale and Oviedo Quality of Sleep Questionnaire were presented.

**Results:**

Patients performed poorer than controls on the Letter Number Sequencing, Arithmetic and Similarities subtests of the Wechsler Adult Intelligence Scale, the Spatial Span subtest of the Wechsler Memory Scale, an N-back task, a verbal fluency task, the Ruff Figural Fluency Test, the Inhibition score of the Stroop Test, the Inhibition and Shifting scores of the Five Digits Test, the Key Search Test and the Zoo Map Task. Moreover, patients exhibited less steep learning curves on the Iowa Gambling Task. Among clinical variables, BMI and pain severity explained the largest proportion of performance variance.

**Conclusions:**

This study demonstrated impairments in executive functions of updating, shifting inhibition, decision making and planning in FMS. While the mediating role of pain in cognitive impairments in FMS had been previously established, the influence of BMI is a novel finding. Overweight and obesity should be considered by FMS researchers, and in the treatment of the condition.

## Introduction

Fibromyalgia syndrome (FMS) is a chronic condition characterized by widespread pain. Frequent accompanying symptoms include morning stiffness, depression, anxiety, fatigue, insomnia and cognitive impairments [1]. Its symptoms severely reduce quality of life and psychosocial functioning [2, 3]. The precise etiology of FMS pain is still unknown, although current models propose that sensitization of central nociceptive pathways plays a key role [4]. Subjectively perceived cognitive problems may include forgetfulness, concentration difficulties, general slowness and language-related deficits [5–7]. Patients' reports indicate that these complaints significantly affect their everyday life and social and professional functioning; thus they are perceived to be among the most serious symptoms of the disease [5, 8–10].

Various studies have used standardized neuropsychological assessment tools to quantify cognitive function in FMS. Reduced performance in affected patients has been observed on tasks indexing working, episodic, semantic. and implicit memory [11–14] as well as selective and sustained attention [12, 15–18]. Other studies have suggested reduced cognitive processing speed [19, 20] and language-related deficits [13, 21]. Even though effect sizes differed considerably among studies and tasks, the available data largely confirms patients' subjective reports of mental disruption, especially in the memory and attention domains.

The present study aimed to investigate higher cognitive abilities, i.e. executive functions, in FMS, which are crucial for completing most everyday tasks. Executive functions enable regulation, co-ordination and sequencing of basic mental operations, thereby adjusting cognition and behavior according to the situational requirement [22]. In his classical taxonomy, Miyake [23] distinguished between three executive function components: “shifting”, “updating” and “inhibition”. The shifting component includes the processes of switching between multiple tasks, operations, or cognitive sets. Updating refers to the monitoring and evaluation of new information, and permanent updating of information stored in working memory. The inhibition function involves the ability to deliberately inhibit or prevent inappropriate automatic responses.

Several earlier studies suggest the presence of executive function impairment in FMS. A study using the Wisconsin Card Sorting Test [24] pointed toward lower performance in the shifting component in FMS patients vs. healthy controls [25]. Some studies showed impairments in updating and inhibition via reading span tasks [26] and the Stroop paradigm [27], respectively [13, 28, 29]. Further research revealed evidence of performance reductions in arithmetic processing tasks [19], decision making [30] and verbal fluency [13, 31, 32], which also place a load on executive functions. However, in other studies, FMS patients did not differ from healthy individuals in their performance on various executive function tasks [20, 33, 34]. Therefore, concluding definitively that there is a generalized deficit in executive functions in FMS would be premature and further research is certainly worthwhile.

While most studies available in the literature were restricted to quantification of single aspects of executive function, the present study used a more comprehensive approach. To this end, a large test battery was compiled systematically to cover the three dimensions of Miyake’s framework. In addition to computerized and paper-pencil tasks quantifying the shifting, updating and inhibition components, decision making and planning tests were applied, which require multiple executive functions and are doubtlessly of high importance in everyday life.

As a secondary aim, the study investigated various clinical factors possibly related to executive function impairment. It has been suggested that an interference effect of pain is implicated in the cognitive impairment seen in FMS [18]. This is supported by the finding of a close association between pain severity and the magnitude of performance decline [9, 11, 13, 25]. It has been argued that in FMS, exaggerated central nervous pain processing detracts from cognition, because it requires enhanced neural resources in brain areas that are involved in both cognition and nociception [11, 18, 19, 35]. Interestingly, in the majority of studies, psychiatric symptoms, including those of depression and anxiety disorders, had a far lower impact on cognitive performance than pain severity, suggesting that these factors only play a subordinate role [11, 16, 18, 19, 25, 35]. Furthermore, it is well-established that sleep disturbance may be accompanied by reduced cognitive performance [36]. Initial evidence suggested that these complaints, which are frequent in FMS, may be involved in patients' cognitive impairment [37].

It may be hypothesized that body weight also plays a role in the cognitive dysfunction that characterizes FMS. Obesity has been shown to be associated with greater symptom severity and reduction of quality of life in FMS [38, 39]. Moreover, studies conducted in overweight and obese individuals drawn from the general population showed inverse associations between body mass index (BMI) and cognitive performance [40–43]. To our knowledge, only a single study has addressed this issue in FMS [44]. While aerobic fitness correlated positively with memory performance, BMI, percent body fat, fat-mass index and waist circumference showed no effect. Nonetheless, on account of the aforementioned findings, and considering the considerable prevalence of overweight in FMS [45], it seemed worthwhile to investigate BMI as another possible factor influencing executive functions.

Finally, possible effects of medication on cognition should be considered. Although a reduction in cognitive impairment due to analgesic medication has been observed [16], most available studies comparing FMS patients using antidepressants, anxiolytics, and opiates and non-opioid analgesics with those not using these drugs did not reveal differences in performance parameters [37, 18, 19, 25].

The principal aim of the study was to compare executive functions between patients with FMS and healthy individuals. In contrast to most previous studies, our research approach allowed differential analysis of performance with respect to shifting, updating and inhibition functions, as well as decision making and mental planning. In addition, possible impacts of clinical pain severity, psychiatric comorbidities, sleep quality, BMI and medication on executive functions were investigated. On account of the findings delineated above, pain symptoms were expected to be more closely related to performance than those of depression and anxiety. While current research suggests only a limited impact of analgesic, antidepressant and anxiolytic medication, a greater degree of sleep disturbance and higher BMI were expected to be associated with poorer task performance.

## Methods

### Participants

A total of 52 FMS patients and 32 healthy control subjects participated in the study. Due to the higher prevalence of FMS in women than men, and to avoid possible gender-related confounding factors, only women were included. Patients were recruited via the Fibromyalgia Association of Jaen and Úbeda (Spain). They were examined by a rheumatologist and met the American College of Rheumatology criteria for FMS [46]. Exclusionary criteria comprised documented head injury, neurological disorders, cardiovascular diseases, metabolic abnormalities, and severe somatic (e.g., cancer) or psychiatric (e.g., schizophrenia, bipolar disorder) diseases. Healthy controls were recruited by means of local advertisements and snowball sampling from among the community. Table 1 displays the sociodemographic and clinical data of both groups. Patients and controls did not differ significantly in terms of age, education level or BMI.

**Table 1:** Demographic and clinical data of the sample; values of t [82] or *x*^*2*^ and p of the group comparison (M = mean, SD = standard deviation).

	FMS patients N = 52	Control group N = 32	t[82] / x^2^	p
**Age (M ± SD)**	51.25 ± 8.67	52.94 ± 6.59	-0.95	.35
**Years of education (M ± SD)**	9.27 ± 3.52	10.59 ± 3.64	-1.65	.10
**BMI (M ± SD)**	28.29 ± 4.49	26.49 ± 4.36	1.80	.075
**Depression (N, %)**	22 (42.30)	2 (6.25)	12.62	< .0001
Anxiety disorder* (N, %)	25 (48.08)	7 (21.88)	5.77	.016
**Antidepressant medication (N, %)** **Anxiolytic medication (N, %)**	27 (51.92) 35 (67.31)	2 (6.26) 8 (25.00)	18.28 14.19	< .0001 < .0001
**Non-opioid analgesic medication (N, %)** **Opiate medication (N, %)**	45 (86.5) 23 (44.2)	8 (25) 1 (31)	32.22 16.40	< .0001 < .0001
**State-Trait Anxiety Inventory: State (M ± SD)**	30.92 ± 11.92	17.19 ± 9.60	5.51	< .0001
**State-Trait Anxiety Inventory: Trait (M ± SD)**	35.29 ± 9.34	17.56 ± 10.24	8.14	< .0001
**Beck Depression inventory (M ± SD)**	21.90 ± 12.56	4.47 ± 5.67	7.39	< .0001
**Fatigue Severity Scale (M ± SD)**	50.56 ± 12.35	19.88 ± 11.14	11.92	< .0001
**Oviedo Quality of Sleep Questionnaire: Insomnia (M ± SD)**	29.73 ± 7.43	17.09 ± 7.52	7.91	< .0001
**Oviedo Quality of Sleep Questionnaire: Hypersomnia (M ± SD)**	8.67 ± 5.01	4.13 ± 1.56	4.98	< .0001
**McGill Pain Questionnaire: Sensory Pain (M ± SD)**	35.59 ± 18.39	12.38 ± 3.85	5.71	< .0001
**McGill Pain Questionnaire: Affective Pain (M ± SD)**	5.92 ± 4.50	0.72 ± 0.71	5.26	< .0001
**McGill Pain Questionnaire: Miscellaneous Pain (M ± SD)** **McGill Pain Questionnaire: Current Pain Intensity (M ± SD)**	9.25 ± 5.90	4.14 ± 2.56	3.81	< .0001
	3.31 ± 0.88	1.48 ± 0.51	8.97	< .0001

* Anxiety disorders comprise panic disorder, generalized anxiety disorder, phobias and adjustment disorder.

### Clinical assessment

The Structured Clinical Interview for Axis I Disorders of the Diagnostic and Statistical Manual for Mental Disorders (SCID) was applied to assess psychiatric disorders [47]. Clinical pain severity was quantified using the Spanish version of the McGill Pain Questionnaire [48]. From this instrument we used three subscales, the sensory, affective, and miscellaneous aspects of pain, in addition to a sum score and a scale for measuring current pain intensity. Symptoms of depression were evaluated by means of the Beck Depression Inventory [49]; the State-Trait Anxiety Inventory [50] was used to evaluate current and habitual anxiety levels. Finally, the Fatigue Severity Scale [51] and the Insomnia, and Hypersomnia scales of the Oviedo Quality of Sleep Questionnaire were presented [52].

### Cognitive tests

The following cognitive tests were primarily aimed at measuring the *updating* function (c.f. Discussion section for detailed discussion of the assignment of the tests to the various types of executive function):

#### Letter number sequencing

This is a subtest of the Wechsler Adult Intelligence Scale, WAIS-III [53] during which the participant is read a sequence of numbers and letters, and recalls the numbers in ascending order and the letters in alphabetical order (21 trials). Performance is indexed by the number of correct responses.

#### Arithmetic

In this WAIS-III subtest, the participant has to mentally solve 20 story-type arithmetic problems within a limited timeframe, ranging between 15 and 120 s. The number of correct solutions indexes performance.

#### Spatial span

In this subtest of the Wechsler Memory Scale, WMS-III [54] a platform including 10 three-dimensional cubes is placed in front of the participant. The experimenter touches several cubes in a predetermined sequence (sequence length, 2 to 9 items); then, the participant has to touch the cubes in the same (forward span), or the reverse (backward span), order (32 trials). In order to limit the number of dependent variables, and because of the correlation between forward and backward span (r = .49 in the total sample), the sum of all correct responses is applied as performance parameter.

#### N-back task [55]

In this test, a continuous stream of letters appears on a computer screen at a constant rate (2 s per letter). The participant is instructed to press one of two keys to indicate whether the presented letter is, or is not, identical to the preceding (1-back condition) or one-to-last (2-back condition) letter. Each condition includes 100 letters; performance is indexed by the number of correct responses (i.e., the sum of correct hits and rejections) in both conditions.

#### Verbal fluency [56]

Here, the participant is asked to produce the largest possible number of words starting with the letters F, A and S, respectively (1 min per letter). The total sum of words indexes performance.

#### Ruff Figural Fluency Test [57]

In this test, five different configurations of dots are presented on a sheet of paper. The participant's task is to draw as many unique designs as possible by connecting the dots in different patterns (time limit = 60 s per configuration). Task performance is given by the total number of original figures produced.

#### Similarities

In this WAIS-III subtest, the participant is read pairs of words representing common objects or concepts, and must indicate how these objects/concepts are similar (analogical reasoning). The number of correct responses indexes performance.

The following tasks primarily assessed the *inhibition* and *shifting* functions:

#### Stroop Test (Color Word Interference Test) [58]

This test consists of four different parts, each containing 50 items. In Part 1 (color naming), the colors of patches have to be named as quickly as possible; in Part 2 (reading) the words red, blue, and green, printed in black, have to be read aloud. In Part 3 (inhibition), these color words are presented in incongruent colors (e.g., the word red written in blue color); the participant is asked to name the color while ignoring the word meaning. Part 4 (shifting) contains the same items as Part 3, but the response mode switches between reading and color naming according to a visual cue. Inhibition performance is the difference in execution time (in s) between the inhibition and color naming conditions (Part 3 vs. Part 1); shifting performance is the time difference between shifting and inhibition (Part 4 vs. Part 3). Lower scores reflect greater inhibition and shifting capacity.

#### Five Digits Test [59]

This task consists of four parts. A series of 50 squares are presented, each of which contains one to five digits or asterisks. In Part 1 (reading), the participant is asked to read the digits as quickly as possible, while in Part 2 (counting), the asterisks have to be counted. In Part 3 (interference), the digits have to be counted. (Note that the numbers of digits in the boxes do not correspond to their arithmetic values.) The requirements of Part 4 (shifting) are identical to those of Parts 1 and 2 (reading, counting), where the task mode changes between trials according to a visual cue. Inhibition is quantified as the time difference (in s) between Part 3 and the mean of Parts 1 and 2; shifting is indexed by the time difference between Part 3 and the mean of Parts 1 and 2. Lower scores reflect greater inhibition and shifting capacity.

#### Wisconsin Card Sorting Test [24]

In this test, the participant must assign a series of 64 cards, displaying different symbols, to one of four reference cards. The cards can be matched by the symbols' number, color or shape. The participant is unaware of the assignment rule (number, color, shape), but receives feedback after each trial (right/wrong). The rule changes after 10 consecutive correct responses, and thus the assignment strategy has to be changed accordingly. Shifting performance was indexed by the percentage of perseverative errors, i.e. incorrect responses that would have been correct for the preceding rule.

A computerized version of the **Iowa Gambling Task** [60] was used for assessment of *decision making* abilities. The task requires the selection of cards from four decks (decks A, B, C, and D) in order to win (virtual) money. Feedback concerning the outcome (gain or loss) is given immediately after each choice. Decks A and B yield high gains and also high losses. If these decks are played continuously a net loss results; thus, they are disadvantageous in the long run. In contrast, decks C and D are associated with small gains and small losses, and result in a net profit if selected continuously; therefore, they are advantageous in the long run. The task was executed across five blocks, each comprising 20 trials. Decision making performance was defined as the difference between the number of advantageous and disadvantageous decisions. This parameter was computed for the entire task, as well as separately for each of the five blocks.

*Planning abilities* were assessed using the **Key Search Test** and the **Zoo Map Task** from the Behavioural Assessment of the Dysexecutive Syndrome [61]. In the Key Search Task, the participant is required to demonstrate how he/she would search for a set of lost keys in a field. In the Zoo Map Task, the participant has to plan a route to visit 6 of 12 possible locations in a zoo. It consists of two parts, i.e. a more demanding open situation, in which little information is provided that would help to generate an appropriate plan, and a situation that involves simply following a concrete, externally imposed strategy. Both planning tests produce scores that are generated according to the functionality and efficacy of the developed strategies.

In addition, the **Revised Strategy Application Test** [62] was applied as a measure of strategic planning and self-regulation. The task includes three simple activities, i.e. figure tracing, sentence copying and object numbering. Activities are presented in two different stacks of 120 items each. Items differ regarding their size (large, small) and time requirements (brief, medium, long). A large items scores 0 points and a small item scores 100 points, where participants are instructed to obtain as many points as possible. The items are intermixed; however, the number of brief items decreases progressively within both stacks. As the execution time of the task is restricted to 10 min, the most efficient strategy is to complete brief items rather than longer ones. Thus, the predisposition to complete items in the presented sequence has to be overcome. Performance is indexed by the number of completed items.

Finally, in order to evaluate possible malingering, the 15-item Rey Memory Test [63] was used. This test is presented to the participant as a difficult memory task, but is actually quite simple because high redundancy among items restricts the information that needs to be remembered. Malingering is indicated by a score below 6.

### Procedure

**Fig 1:**
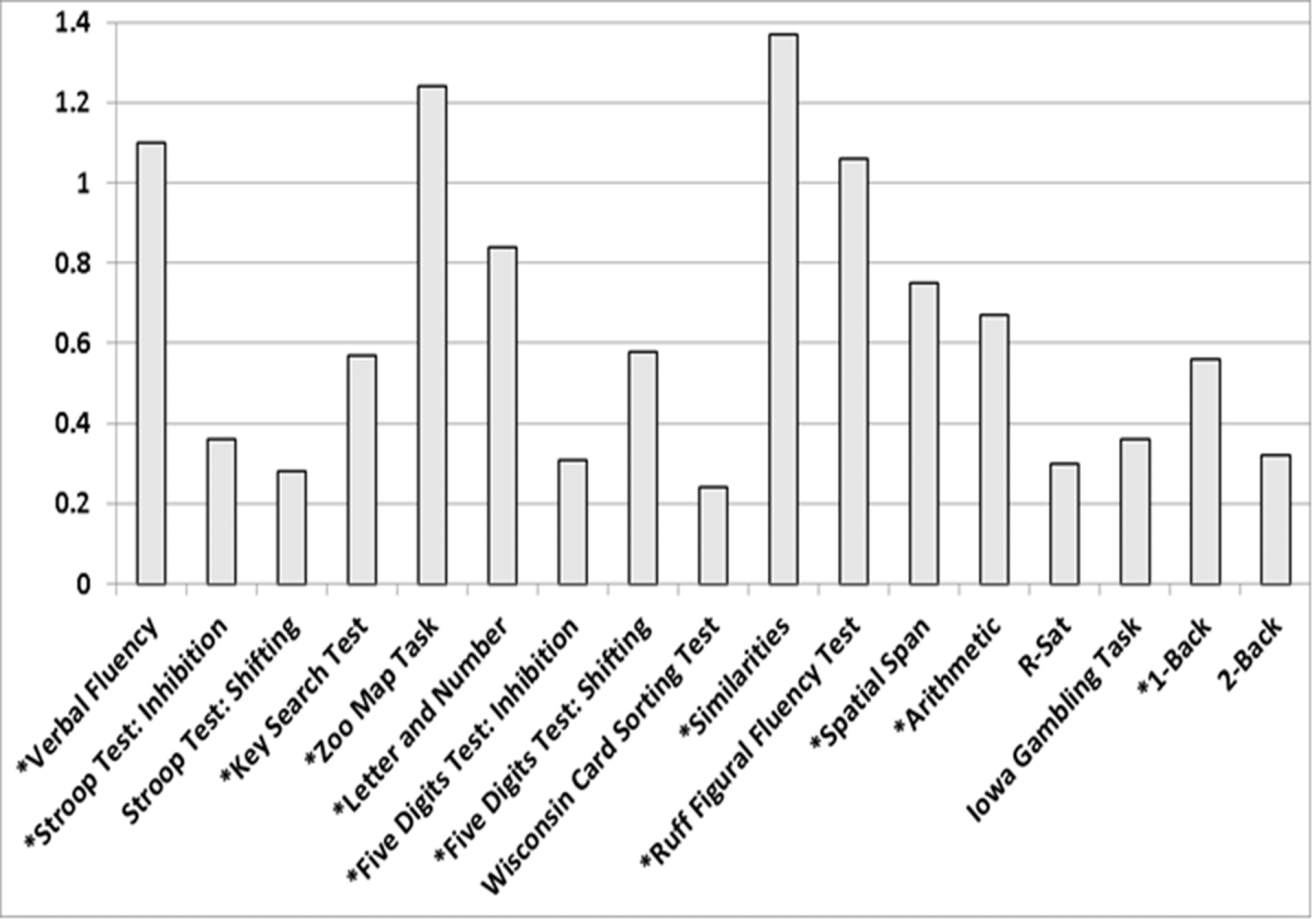
Values of Cohen's d for the group comparisons in task parameters (*variables in which the group factor was significant).

All participants were tested individually within two sessions lasting approximately 2 hours each (including breaks). In the first session, a clinical psychologist took the patients’ clinical history, recorded sociodemographic data and medication use, and evaluated possible violations of the exclusionary criteria. After that, SCID interviews were conducted and the clinical questionnaires were presented. In addition, participants completed the 15-item Rey Memory Test. None of the participants met the criteria for malingering. Cognitive testing was carried out in the second session. The tests were presented in a fixed order, alternating between verbal and non-verbal tasks, and between more and less demanding tasks (c.f. Fig 1 for test sequence). The study was approved by the Ethics Committee for Human Research of the University of Jaén and all participants provided written informed consent.

### Statistical analysis

Group differences in executive functions were analyzed by multivariate analysis of variance (MANOVA). Dependent variables comprised all parameters of test performance. Age, educational level and BMI were included as covariates in this model. An additional two-way analysis of variance (ANOVA) was computed for the Iowa Gambling Task, including the between-subjects factor of group (FMS patients vs. controls) and the within-subjects factor of task block (blocks 1 to 5). Effects of medication use and comorbid psychiatric disorders were tested by performing stratified analyses in the FMS group through MANOVA models that compared patients using and not using each type of medication (done separately for antidepressants, anxiolytics, non-opioid analgesics, and opiates) and comparing patients suffering and not suffering from depression and anxiety disorders. Effect sizes are indicated by adjusted eta squared (η^2^_p_) and Cohen's d.

Associations between clinical variables and executive functions were analyzed in two steps, both restricted to the FMS group (N = 52). Firstly, as an exploratory analysis, Pearson correlations were computed between clinical variables and performance parameters. Secondly, multiple regression analyses were computed. Two blocks were entered in the analyses: (1) age and years of education (simultaneously, enter method) and (2) BMI and clinical variables (stepwise method). The adjusted R^2^ is presented for the change in prediction associated with each new block. A maximum number of five predictor variables was entered in these analyses (a ratio of at least 10 cases per predictor is considered appropriate [64]).

## Results

### Group comparisons

The MANOVA comparing the FMS patients and healthy controls revealed a significant group effect (F[17,63] = 4.867 p < .0001, η^2^_p_ = .57). Furthermore, significant effects of the covariates BMI (F[17,63] = 2.13, p = .016, η^2^_p_ = .36) and educational level (F[17,63] = 5.17, p < .0001, η^2^_p_ = .58) arose. Table 2 displays the means and standard deviations of the performance parameters, as well as the statistics of the univariate group comparisons, and the effects of BMI on performance parameters. Significant group differences arose for all performance parameters except for the 2-back condition of the N-back Task, the Shifting index of the Stroop Test, the Wisconsin Card Sorting Test, the total score of the Iowa Gambling Task and the Revised Strategy Application Test. All group differences suggested poorer task performance in FMS patients than controls. Values of Cohen's d for the group comparisons are displayed in Fig 1. Significant effects of BMI were observed for the Letter Number Sequencing, Arithmetic and Similarities tests, both scores of the Five Digits Test, the Inhibition index of the Stroop Test, the Wisconsin Card Sorting Test, the total score of the Iowa Gambling Task and the Revised Strategy Application Test. Higher BMI was associated with poorer performance in all task parameters.

**Table 2:** Indices of test performance: Means (M) and standard deviations (SD) for FMS patients and healthy controls; values of F, p and η^2^_p_ for the effects of group and the covariate body mass index (BMI).

Test	FMS Patients N = 52 M SD		Control group N = 32 M SD		F [1,82] (group)	p (group)	η^2^_p_ (group)	F[1,82] (BMI)	p (BMI)	η^2^_p_ (BMI)
**Letter Number Sequencing**	7.31	2.60	9.63	2.92	14.33	< .0001	.15	9.01	< .01	.10
**Arithmetic**	9.33	3.05	11.41	3.18	8.90	< .01	.10	5.70	.019	.065
**Spatial Span**	12.35	2.85	14.47	2.79	11.16	< .0001	.12	0.63	.43	< .01
**N-back task (1-back)**	92.23	12.05	97.22	4.32	5.10	.027	.060	2.48	.12	.03
**N-back task (2-back)**	80.54	9.30	83.23	10.23	1.54	.22	.018	1.20	.28	.01
**Verbal fluency**	24.75	10.99	36.69	10.76	23.74	< .0001	.22	2.49	.12	.029
**Ruff Figural Fluency Test**	50.33	19.15	69.78	17.45	21.85	< .0001	.21	3.31	.072	.039
**Similarities**	13.79	4.94	21.06	5.66	38.43	< .0001	.32	7.60	< .01	.085
**Stroop Test: Inhibition**	35.33	16.34	23.97	12.67	11.27	< .0001	.12	7.72	< .01	.086
**Stroop Test: Shifting**	13.69	23.07	8.25	14.09	1.45	.23	.017	0.84	.36	.010
**Five Digits Test: Inhibition**	20.96	13.26	13.59	8.79	7.76	< .01	.086	8.21	< .01	.091
**Five Digits Test: Shifting**	36.48	18.46	26.69	15.32	6.32	.014	.072	15.12	< .0001	.16
**Wisconsin Card Sorting Test**	22.65	12.04	19.38	13.40	1.13	.29	.014	4.59	.026	.059
**Iowa Gambling Task**	7.10	24.12	16.46	28.10	2.63	.11	.031	5.28	.024	.061
**Key Search Task**	7.60	3.33	9.59	3.81	6.38	.013	.072	2.61	.11	.031
**Zoo Map Test**	11.29	3.52	14.63	1.48	25.83	< .0001	.24	2.75	.10	.032
**Revised Strategy Application Test**	83.29	15.14	87.59	11.93	1.87	.18	.022	7.90	< .01	.088

**Fig 2:**
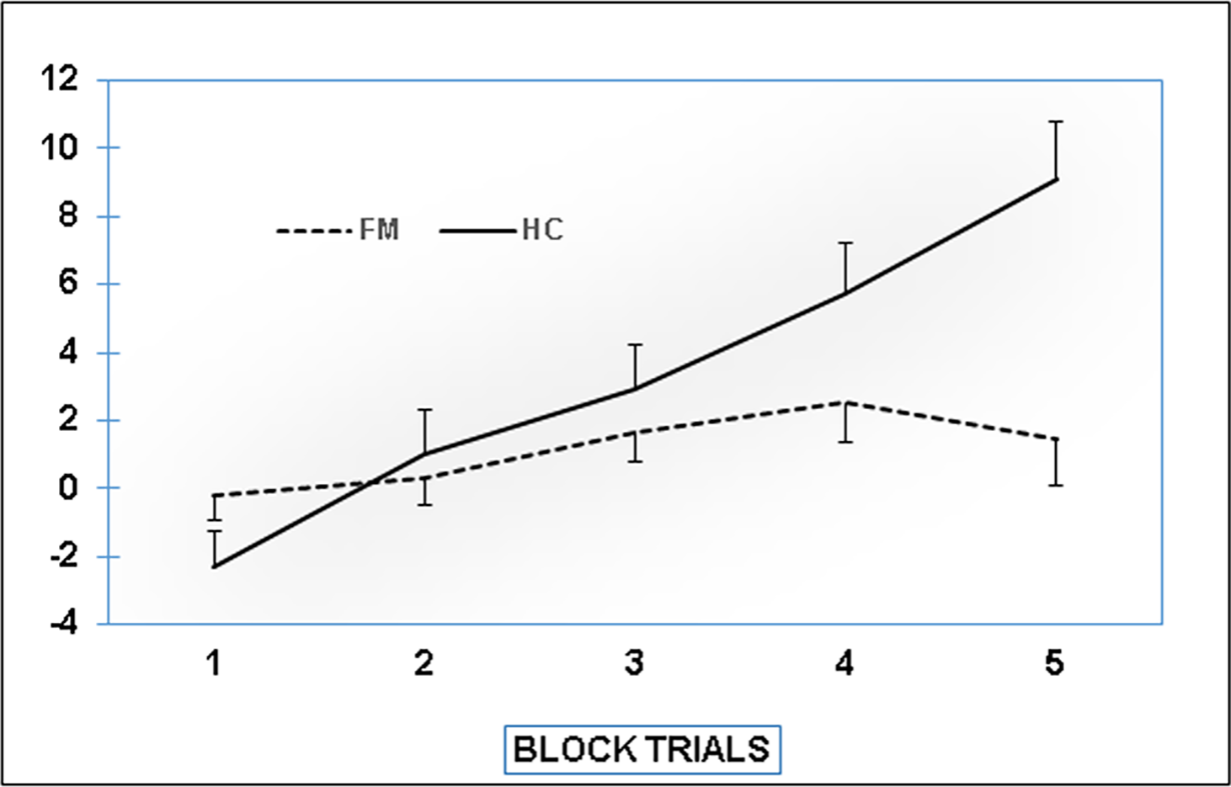
Performance on the Iowa Gambling task (differences between advantageous and disadvantageous decisions for the five blocks of task execution).

Performance in the Iowa Gambling Task across the five task blocks is displayed in Fig 2. The ANOVA did not reveal a main effect of group (F[1,82] = 3.37, p = .070, η^2^_p_ = .039); however, both the block factor and the interaction were significant (block: F[1,82] = 33.95, p < .0001, η^2^_p_ = .29; interaction: F[1,82] = 15.06, p < .0001, η^2^_p_ = .16). Post-hoc testing was performed as follows: to analyze performance changes across blocks in FMS patients and controls, one-way analyses were conducted separately for both groups. In addition, performance in each of block was compared between groups using t-tests. One-way analyses indicated a significant performance increase across the five blocks in controls F[4,28] = 9.35, p < .0001, η^2^_p_ = .57), but not in patients (F[4,48] = 1.47, p = .23, η^2^_p_ = .11). Post-hoc comparisons between groups reached significance only for block 5 (t[82] = -3.57, p < .01 for block 5; ts [82] < 1.67, ps > .10 for the remaining blocks).

The MANOVAs comparing executive function between FMS patients with and without depression and anxiety disorders (from SCID) did not reveal significant results (depression: F[17,31] = 0.87, p = .61, η^2^_p_ = .32; anxiety disorders: F[17,31] = 0.65, p = .83, η^2^_p_ = .26). This was also true for the models concerning the use of anxiolytics (F[17,31] = .55, p = .90, η^2^_p_ = .23), analgesics (F[17,31] = .34, p = .99, η^2^_p_ = .16), opiates (F[17,31] = .92, p = .56, η^2^_p_ = .33) and antidepressants (F[17,31] = 1.94, p = .053, η^2^_p_ = .52). In all stratified analyses, significant effects of the covariates BMI (Fs between 2.28 and 2.40, ps < .030, η^2^_p_ between .55 and .57) and educational level (Fs between 2.72 and 3.02, p < .01, η^2^_p_ between .60 and .62) arose.

### Associations between clinical variables and executive function

#### Correlation analysis

Results of the exploratory correlation analysis are presented in Table 3. Performance on the 1-back condition of the N-back task correlated negatively with the McGill Pain Questionnaire scale of Sensorial Pain; performance on the 2-back condition correlated negatively with the Affective Pain scale. Scores of the Letter Number Sequencing, and Similarities tasks correlated negatively with BMI. Furthermore, BMI correlated positively with the Inhibition score of the Stroop Test, the Inhibition and Shifting scores of the Five Digits Test, and the percentage of perseverative errors on the Wisconsin Card Sorting Test (note that positive correlations reflect inverse associations between BMI and task performance). Perseverative errors also correlated with the McGill Pain Questionnaire scale of Sensorial Pain. Finally, performance on the Iowa Gambling Task (sum score of the five blocks) correlated negatively with the Current Pain Intensity score of the McGill Pain Questionnaire, the Fatigue Severity Scale score, and the Hypersomnia score of the Oviedo Quality of Sleep Questionnaire.

**Table 3:** Correlations between clinical variables and body mass index and cognitive test scores in FMS patients.

Test	Trait anxiety (STAI)	State anxiety (STAI)	Depression (BDI)	Fatigue (FSS)	Insomnia (COS)	Hypersomnia (COS)	Sensory Pain (MPQ)	Affective pain (MPQ)	Miscellaneous Pain (MPQ)	Current Pain Intensity (MPQ)	BMI
**Letter Number Sequencing**	-.02	.15	.19	.16	.17	-.01	-.05	-.06	-.06	-.07	-.31*
**Arithmetic**	.11	.11	.11	.11	.20	-.04	-.07	.03	-.04	.06	-.21
**Spatial Span**	-.15	-.03	.05	-.01	-.02	-.14	-.20	-.13	-.15	-.20	-.07
**N-back task (1-back)**	-.13	-.03	-.13	.25	.19	.06	-28*	-.09	-.18	.09	-.05
**N-back task (2-back)**	.08	.04	.03	-.04	.25	-.16	-.23	-.36**	-.25	.04	.04
**Verbal fluency**	.09	.03	.01	.15	.04	-.03	-.20	-.27	-.22	-.17	-.15
**Ruff Figural Fluency Test**	.04	-.11	-.01	.12	.22	.04	-.06	-.06	.01	.04	-.14
**Similarities**	-.01	.08	.09	.27	.12	-.08	-.19	-.13	-.23	-.05	-.35*
**Stroop Test: Inhibition**	.20	.23	.13	-.27	-.07	.05	.11	.13	.10	.12	.38**
**Stroop Test: Shifting**	-.05	-.07	-.10	.14	.04	-.04	.03	.04	-.09	-.02	.08
**Five Digits Test: Inhibition**	.01	.12	.04	-.07	-.07	.13	.13	.04	.12	.18	.31*
**Five Digits Test: Shifting**	-.02	.02	-.11	-.19	-.04	.04	.19	.15	.20	.14	.51**
**Wisconsin Card Sorting Test**	-.20	-.15	-.24	-.18	-.18	.03	.30*	.08	.25	.08	.32*
**Iowa Gambling Task**	-.22	-.10	-.18	-.36*	-.02	-.27*	-.17	-.18	-.13	-.27*	-.14
**Key Search Task**	.05	-.06	-.16	.01	.19	-.03	-.05	-.01	.03	.04	-.15
**Zoo Map Test**	.01	.04	.21	.18	.11	-.00	.08	.04	-.06	.09	-.17
**Revised Strategy Application Test**	-.06	-.18	-.05	-.13	-.08	.14	.07	.01	.11	.04	-.14

Note

** p < 0.01

* p < 0.01, two-tailed testing.

#### Regression analysis

Table 4 includes the results of the regression analysis for the prediction of test performance from clinical variables (only significant results of the second block are indicated). The regression model computed for the 2-back condition of the N-back task revealed the Affective Pain scale of the McGill Pain Questionnaire to be a significant predictor. The Inhibition score of the Stroop Test was predicted by BMI in a first regression model; in a second regression model, Trait Anxiety (from the State-Trait Anxiety Inventory) also reached significance. In further regression analyses, the Inhibition and Shifting scores of the Five Digits Test were predicted by BMI. Performance on the Iowa Gambling Task (sum score of the five blocks) was predicted by the Fatigue Severity Scale score; and perseverative errors on the Wisconsin Card Sorting Test were predicted by the Sensorial Pain score of the McGill Pain Questionnaire. The regression model computed for the 1-back condition of the N-back task revealed a marginal effect of the Sensorial Pain scale of the McGill Pain Questionnaire (β = -.26, r^2^ = 0.07, t = -1.91, p = .063). Age and years of education were controlled for in each model. Note that the positive *β*-weights in the models for the Stroop Test, Five Digits Test and Wisconsin Card Sorting Test reflect inverse relationships between task performance and the clinical variables.

**Table 4:** Regression analysis for the prediction of cognitive test scores from clinical variables and body mass index in FMS patients (only significant results of the second block are presented).

Test	Model	Predictor	Β	r^2^	t	p
**N-back task (2-back)**	Model 1	McGill Pain Questionnaire: Affective Pain	-.33	0.11	-2.56	.014
**Five Digits Test: Inhibition**	Model 1	Body Mass Index	.36	0.10	2.39	.021
**Five Digits Test: Shifting**	Model 1	Body Mass Index	.47	0.18	3.54	.001
**Stroop Test: Inhibition**	Model 1 Model 2	Body Mass Index	.38	0.12	2.72	.009
		Body Mass Index	.39	0.07	2.92	.005
		State-Trait Anxiety Inventory: Trait	.28		2.15	.037
**Wisconsin Card Sorting Test**	Model 1	McGill Pain Questionnaire: Sensorial Pain	.29	0.08	2.32	.024
**Iowa Gambling Task**	Model 1	Fatigue Severity Scale	-.37	.14	-2.77	.008

Note: Standardized β and adjusted r^2^ change are indicated.

## Discussion

The study explored executive functions in FMS patients using a comprehensive test battery assessing the dimensions of updating, shifting and inhibition, as well as decision making and mental planning. Patients performed poorer than healthy individuals on the Letter Number Sequencing, Arithmetic and Similarities subtests of the Wechsler Adult Intelligence Scale, the Spatial Span subtest of the Wechsler Memory Scale, the 1-back condition of an N-back task, the Ruff Figural Fluency Test, and a verbal fluency task. A performance reduction was also observed on the Inhibition score of the Stroop Test, the Inhibition and Shifting scores of the Five Digits Test, and the Key Search Test and Zoo Map Task of the Behavioral Assessment of the Dysexecutive Syndrome. In addition, a less steep learning curve on the Iowa Gambling Task was seen in patients versus controls. Some of the test scores exhibited linear associations with clinical variables including pain severity, fatigue, hypersomnia, trait anxiety and BMI.

The updating dimension of executive functions was addressed by the Letter Number Sequencing, Spatial Span and N-back tasks. As defined in Miyake's framework, these tasks require active tracking, manipulation and permanent retention of information in working memory [23]. Cognitive fluency, such as assessed by the verbal fluency task and Ruff Figural Fluency Test, may also be discussed in the context of updating. It has been argued that in this type of task, instructions and earlier responses have to be maintained in working memory in order to constantly create new, original responses [65]. Consistent with this, it has been reported that among various cognitive tasks, an operation span task measuring the updating function was most closely associated with performance in letter and category fluency tests [66]. The applied arithmetic task, during which verbal and numerical information had to be sustained in working memory and utilized for the required mathematical operations, may also place a considerable load on the updating component. Analogical reasoning, as required in the Similarities task, cannot be unambiguously assigned to any of the components of executive function. However, it has been shown that in addition to general problem solving, successful completion of such tasks strongly depends on working memory updates [67–69]. Taken together, our observations support the notion of markedly impaired updating performance in FMS. This is in accordance with a number of earlier reports. FMS patients performed more poorly on a reading span task [16, 26], as well as the working memory subtest of the Test of Everyday Attention [70]. In previous studies FMS patients scored lower on tasks assessing at computation and reading span [71] and on a serial addition test [72].

Our findings regarding the shifting component of executive functions were less consistent. While patients exhibited lower values on the Shifting index of the Five Digits Test, no group differences arose in the same index according to the Stroop Test, and in terms of perseverative errors on the Wisconsin Card Sorting Test. As both the Five Digits Test and Stroop Test quantify task switching ability in a similar manner, the divergence between the tests is somewhat surprising. Previous studies using the Wisconsin Card Sorting Test in FMS revealed controversial results. A reduced percentage of non-perseverative errors in patients vs. controls, but no differences in perseverative errors or failures to maintain set were reported [25]. Other studies did not reveal group differences in any of the test parameters [28]. It is believed that among the available indices, perseverative errors best reflect mental flexibility and thus the shifting dimension [73]. Nonetheless, the expression of this parameter also varies subject to the ability to deduce rules and thus engage in abstract thinking. While a negative finding revealed by the Trail Making Test [74] challenges the notion of shifting impairments in FMS [28], further research is warranted to achieve clarity regarding this component.

The results pertaining to the Inhibition parameters of the Stroop Test and the Five Digits Test support the notion of FMS patients having a diminished ability to deliberately override dominant or automatic responses. The reduced inhibition performance on the Stroop Test replicated an earlier finding [28]. It has been argued that intact inhibitory processes are also essential in fluency tasks during which the participant must suppress inadequate responses and repetitions [65, 66]. The relevance of this to analogical reasoning has also been pointed out, where efficient suppression of task-irrelevant information is necessary [67, 68]. The performance reduction in the verbal and figural fluency tests and the Similarities task may thus also partly reflect inhibition impairment. Although contrary findings have been reported [20, 33], reduced inhibitory capacity is likely in FMS.

The response pattern on the Iowa Gambling Task also differed between the groups in this study. While task performance, defined as the difference between the number of advantageous and disadvantageous decisions, strongly increased during performance of the task in healthy individuals, only a slight and non-significant increase arose in patients. This finding is in line with earlier reports of an aberrant learning curve in this task, suggesting compromised decision making and slower learning progress in FMS [25, 30]. The Iowa Gambling Task was developed within the framework of the somatic marker hypothesis, according to which intuitive decision making is guided by information arising from within the body [60, 75]. It has been shown that poor access to bodily signals (i.e. interoceptive sensibility) is associated with altered decision making, reflected in reduced performance on the Iowa Gambling Task [76]. Interoceptive sensibility is commonly quantified as the ability to perceive one’s own heartbeat, a metric on which individuals vary substantially [77, 78]. Interestingly, patients with FMS displayed markedly reduced cardiac interoceptive sensitivity [79]. Evidence of impaired tactile perception points toward a more general impairment in somatic information processing in FMS [80, 81]. Considering the relevance of bodily perception to decision making, it can be hypothesized that these deficits may contribute to the corresponding impairments in FMS [82, 35, 79].

To our knowledge, mental planning in FMS was addressed for the first time in this study. Patients performed markedly more poorly on the Key Search Test and Zoo Map Task of the Behavioral Assessment of the Dysexecutive Syndrome, which were designed as behavioral measures of planning abilities that constitute an important aspect of executive function [61]. Several evaluation studies on these tests have supported their construct validity, while it has been claimed that their ecological validity is superior to that of standard executive function tests [61, 83], although some divergent results also exist [84]. No group difference was found in the Revised Strategy Application Test, which assessed strategic planning and self-regulation. Nonetheless, as the relevance of the abilities quantified by these tests to everyday life is beyond question, further research on this issue is clearly warranted.

As a secondary aim, the study investigated the implications of various clinical features for executive function impairment in FMS. Among the questionnaire scales, those of the McGill Pain Questionnaire were most closely associated with cognitive parameters. The scales reflecting sensory and affective pain experience correlated negatively with performance on the 1- and 2-back conditions of the N-back task, respectively. Sensory pain was also inversely related with performance on the Wisconsin Card sorting test, while current pain intensity correlated with performance on the Iowa Gambling Task. Regression analysis controlling for demographic variables confirmed the inverse association between sensory pain and the 1-back condition of the N-back task, and between affective pain and the 2-back condition. In addition, sensory pain experience predicted Wisconsin Card Sorting Test performance. These findings are in accordance with earlier studies on the especial importance of pain symptoms to cognitive decline in various domains. For example, in an FMS study on processing speed, verbal fluency and memory recall and reproduction, pain severity was closely related to performance decline [13]. In further studies, self-reported clinical pain predicted performance on tasks measuring sustained attention, arithmetic performance, and working and implicit memory [11, 16, 19]. In the field of executive functions, the expression of pain symptoms correlated with several performance parameters of the Iowa Gambling Task and the Wisconsin Card Sorting Test [25]. Pain may interfere with cognition by requiring enhanced neural resources in relevant brain structures. The brain networks underlying pain processing and executive function partially overlap. A neuromatrix of nociception has been identified, including the somatosensory cortex, anterior cingulate, insula and thalamus, as well as the prefrontal cortex [85]. The prefrontal cortex is of particular relevance in the present context, because it plays a key role in executive function [23]. It is well-established that exaggerated activity of the central-nervous pain matrix is implicated in the hyperalgesia that characterizes FMS [4]. According to neuroimaging studies, this hyperactivity is also present in prefrontal areas [4, 86, 87]. This implies increased demands on prefrontal areas, and thus reduced processing resources for executive function. Regarding brain metabolism, catecholaminergic neurotransmission may be relevant in this regard. Dopamine is involved in pain inhibition and reduced dopaminergic activity has been demonstrated in FMS [88, 89]. Dopamine, in turn, is an essential transmitter for executive function processing in prefrontal areas [90].

Psychiatric comorbidity, especially symptoms of depression and anxiety, may also contribute to the mediation of executive function impairments in FMS. However, except for the negative correlation between Trait Anxiety and the Inhibition index of the Stroop Test, the Beck Depression Inventory and State-Trait Anxiety Inventory scores did not correlate with any of the performance parameters. In addition, the stratified analysis comparing FMS patients diagnosed and not diagnosed with comorbid affective and anxiety disorders did not yield significant results, suggesting that these symptoms had no discernable impact on executive function. This is surprising insofar as it is well-known that both depression and anxiety disorders may be accompanied by executive function impairments [91, 92]. Moreover, it is has been documented that in healthy individuals, aversive affective states may interfere with optimal executive function [93, 94]. Nonetheless, the present result is in accordance with some earlier studies indicating that psychiatric comorbidity has relatively little impact on cognition in FMS. In stratified analyses similar to those conducted in the current study, FMS patients with categorically diagnosed depression and anxiety disorders did not differ from those not diagnosed with these conditions in tasks measuring attention [18], arithmetic abilities [37] and implicit memory [11] (however, c.f. [16] for an example of reduction in arithmetic performance in FMS patients with comorbid anxiety disorders). Depression and anxiety symptoms assessed via questionnaire scales had markedly less impact on attention [18], arithmetic abilities [19], and implicit memory [11] than pain severity. In addition, measures of affective distress did not correlate with impairments in tasks of attention and working memory [16], nor with executive functions [25] in FMS.

A bias related to medication is also unlikely given that executive function did not differ between MFS patients using and not using antidepressants, anxiolytics, analgesics or opiates. Again, this is in line with earlier studies suggesting only a minor role of medication in FMS patients' cognitive impairment. For example, stratified analysis comparing FMS patients using and not using antidepressants, anxiolytics, non-opioid analgesics or opiates did not reveal differences in attention, implicit memory or arithmetic performance [11, 18, 37]. A lack of impact of pharmacological treatment on FMS patients' performance on the Wisconsin Card Sorting Test, and an only minor impact on their performance on the Iowa Gambling task, were also reported [25]. Interestingly, even better performance among patients using stable doses of opiates versus those not taking this medication was reported for auditory working memory [16] and arithmetic performance [19]. In a similar vein, follow-up studies of pain patients receiving opiate treatment demonstrated slight enhancement of executive function [95] and augmentation of evoked EEG potentials, possibly reflecting improvement of perceptual-cognitive status [96]. The latter findings clearly contradict the assumption of cognitive impairment due to morphine-induced sedation. Altogether, it may be concluded that it is very unlikely that cognitive impairment in FMS occurs as a result of pharmacological treatment.

Sleep disorders and fatigue may also be implicated in the cognitive deficits seen in FMS. The total score of the Iowa Gambling Task was inversely related with the Fatigue Severity Scale score and the Hypersomnia scale score of the Oviedo Quality of Sleep Questionnaire. Although associations were restricted to this single performance parameter, they nevertheless point toward possible involvement of these concomitant symptoms in executive dysfunction [16, 37]. This is supported by correlations between fatigue levels and cognitive impairment in other patient groups, including those with cancer [97], multiple sclerosis [98] or myasthenia gravis [99]. In a similar vein, concentration and memory problems have also been reported in individuals suffering from insomnia [36] and chronic fatigue [100, 101].

Finally, the study tested the hypothesis of a role for body weight in mediating executive function impairments in FMS. This hypothesis received strong support from the MANOVA comparing FMS patients and controls. Univariate tests showed significant effects of BMI on the Letter Number Sequencing, Arithmetic and Similarities tests, the Inhibition and Shifting scores of the Five Digits Test, the Inhibition index of the Stroop Test, the Wisconsin Card Sorting Test, the total score of the Iowa Gambling Task and the Revised Strategy Application Test. The effects of BMI were also significant in all stratified analyses pertaining to the subgroup comparisons. In addition, correlation and regression analyses demonstrated inverse linear relationships between BMI and performance on a large proportion of the tasks. This observation is important insofar as overweight and obesity are seen frequently in FMS. For example, Okifuji et al. [45] reported that 50% of patients in a US American FMS sample were obese, and an additional 21% were overweight. Mean BMI in the present FMS sample was 28.29 kg/m^2^, which is in the upper range of pre-obesity. Executive dysfunction in obesity is well-known [40–43]. A very recent study demonstrated lower performance in obese individuals in tasks assessing inter alia conceptualization, inhibition, problem solving, verbal fluency and cognitive flexibility [40]. Another study demonstrated poorer performance of pre-obese and obese individuals on a modified Stroop test, the Trail Making Tests and a visuospatial learning task [41]. Overweight and obesity may thus constitute another pathway linking FMS with impaired cognition. Even though somewhat speculative, there may be a role for dopamine metabolism in this regard. Dopaminergic pathways are implicated in the regulation of eating behaviors [102]; obesity was associated with reduced dopamine metabolism in the cingulate, as well as the orbital and dorsolateral prefrontal cortex [103]. As initially stated, dopamine is essential to executive function [90]; and reduced dopaminergic neurotransmission may be involved in the impaired pain inhibition, and thus hyperalgesia, characterizing FMS [88, 89]. On a behavioral level, low physical activity may be an important factor. As a major cause of overweight and obesity, sedentarism is prevalent in FMS [104]. On the other hand, cross-sectional as well as intervention studies demonstrated that higher levels of physical activity are associated with increased performance in executive function tasks [105, 106, 107]. These considerations may be of crucial relevance in FMS treatment, where increasing physical activity, particularly aerobic training, is acknowledged to be among the most beneficial methods [104, 108].

Some methodological limitations of the study warrant discussion. At first, the applied test battery did not include a specific measure of attentional performance. Therefore, we cannot estimate the degree to which the patients' performance reductions in the executive function tests can be ascribed to deficits in basic attentional functions. Another restriction pertains to the lack of any assessment of the mental effort invested in the tasks. Although the application of the 15-item Rey Memory Test did not reveal evidence of malingering in any of the participants, a contribution of lower effort in FMS patients to the group differences in performance cannot be ruled out [109, 110]. Moreover, tasks were presented in a fixed rather than random order. Therefore, in interpreting these findings, effects of task sequence, especially on the occurrence of fatigue during the final tasks, cannot be ruled out. However, the effect sizes of the group differences seemed to be independent of the position in the task sequence (see Fig 1); as such, it is unlikely that the greater levels of fatigue in patients than controls influenced the results. Regarding the possible mediation of executive dysfunction by overweight, assessment of additional physical and behavioral variables may be informative in future studies. For example, assessments of physical activity, aerobic fitness, waste circumference or body fat may be help to uncover the underlying mechanisms [44].

In conclusion, this study included the most comprehensive analysis of executive function in FMS done so far. While it added weight to the notion of performance impairments in the updating and inhibition components, the state of research still does not allow for firm conclusions to be drawn regarding the shifting function. The study furthermore corroborated earlier findings of compromised decision making in affected patients, and provided the first evidence of poorer planning abilities. Regarding clinical factors mediating these impairments, it confirmed the view that pain experience plays a more important role than accompanying symptoms like depression, anxiety, fatigue or sleep disorders. It addition, a marked involvement of overweight and obesity in executive dysfunction is likely, and thus should receive more attention in future research.
